# Molecular Characterization of the Region 7q22.1 in Splenic Marginal Zone Lymphomas

**DOI:** 10.1371/journal.pone.0024939

**Published:** 2011-09-21

**Authors:** Cristina Robledo, Juan L. García, Rocío Benito, Teresa Flores, Manuela Mollejo, José Ángel Martínez-Climent, Eva García, Norma C. Gutiérrez, Miguel A. Piris, Jesús M. Hernández

**Affiliations:** 1 Unidad de Diagnóstico Molecular y Celular del Cáncer, IBMCC, Centro de Investigación del Cáncer, Universidad de Salamanca-CSIC, Salamanca, Spain; 2 Instituto de Estudios de Ciencias de la Salud de Castilla y León, Salamanca, Spain; 3 Servicio de Patología, Hospital Universitario de Salamanca, Salamanca, Spain; 4 Servicio de Patología, Hospital Virgen de la Salud, Toledo, Spain; 5 Área de Oncología, Centro de Investigación Médica Aplicada, Universidad de Navarra, Pamplona, Spain; 6 Unidad de Genómica, Centro de Investigación del Cáncer, Universidad de Salamanca-CSIC, Salamanca, Spain; 7 Servicio de Hematología, Hospital Universitario de Salamanca, Salamanca, Spain; 8 Centro Nacional de Investigaciones Oncológicas (CNIO), Madrid, Spain; Instituto Nacional de Câncer, Brazil

## Abstract

Splenic marginal zone lymphomas (SMZL) are an uncommon type of B-cell non-Hodgkin's lymphoma (NHL-B) in which no specific chromosomal translocations have been described. In contrast, the most frequent cytogenetic abnormality is the loss of the long arm of chromosome 7 (7q). Previous reports have located this loss in the 7q32 region. In order to better characterize the genomic imbalances in SMZL, molecular studies were carried out in 73 patients with SMZL. To gain insight into the mapping at 7q a tiling array was also used. The results confirmed the loss of 7q as the most frequent change. In addition, several abnormalities, including 4q22.1, 1q21.3–q22, 6q25.3, 20q13.33, 3q28, 2q23.3–q24.1 and 17p13, were also present. A loss of 7q22.1 at 99925039–101348479 bp was observed in half of the cases. The region of 7q22.1 has not previously been characterised in SMZL. Our results confirmed the presence of a new region of loss on chromosome 7 in these NHL.

## Introduction

Splenic marginal zone lymphomas (SMZL) are low-grade B-cell lymphomas with a micronodular pattern of spleen involvement, occupying the marginal zone [1]. In the Revised European-American Classification of Lymphoid Neoplasm (REAL), SMZL is considered as a provisional entity, and is included with marginal zone lymphoma of mucosa-associated lymphoid tissue (MALT) type and nodal marginal zone lymphoma in the class of marginal zone lymphomas [2]. However, in the World Health Organization classification, SMZL is regarded as a separate entity [3]. SMZL accounts for fewer than 1% of the non-Hodgkin's B-cell lymphomas (NHL-B).

Cytogenetic abnormalities are commonly present in SMZLs. The most frequent of these are deletions on 7q (30–40%) and gains of 3q (20–30%) and 12q (15–20%). Complex chromosomal imbalances are also common [4]. Losses on 7q mainly involve band 7q32, although distinct regions of loss located either centromerically or telomerically to this region have also been identified [5]–[12]. Upon analyzing SMZL by means of chromosome-based comparative genomic hybridization (CGH) the most frequent chromosomal numerical imbalances proved to be gains of 3q (25%), 5q (28%), 9q (21%), 12q and 20q (22%), and losses of 7q (25%), 6q (20%), 14q (10%), and 17p (10%) [5], [13]–[16]. Using interphase fluorescence *in situ* hybridization (FISH), microsatellite LOH analysis, and chromosome-based CGH analysis, several studies have mapped the common region of the 7q deletion in SMZL to 11.4 Mb at 7q31.3–7q33 [9], [17]. By contrast, the information about the presence of gains and losses of chromosomes obtained from array-based comparative genomic hybridization (CGH arrays) is scarce and comprised only of small series. These have not contributed to the further delineation of the minimal common region of the 7q deletion [18]–[21].

In the present study, a large series of SMZL was analyzed by CGH arrays, followed by a high-resolution chromosome 7 tiling array. The results were confirmed by molecular studies, to characterize the minimal common region of the 7q deletion. Our results identify new regions involved in this disease, and characterize the losses on 7q22.1 as a common molecular abnormality in SMZL.

## Results

### BAC CGH array

**Figure 1:**
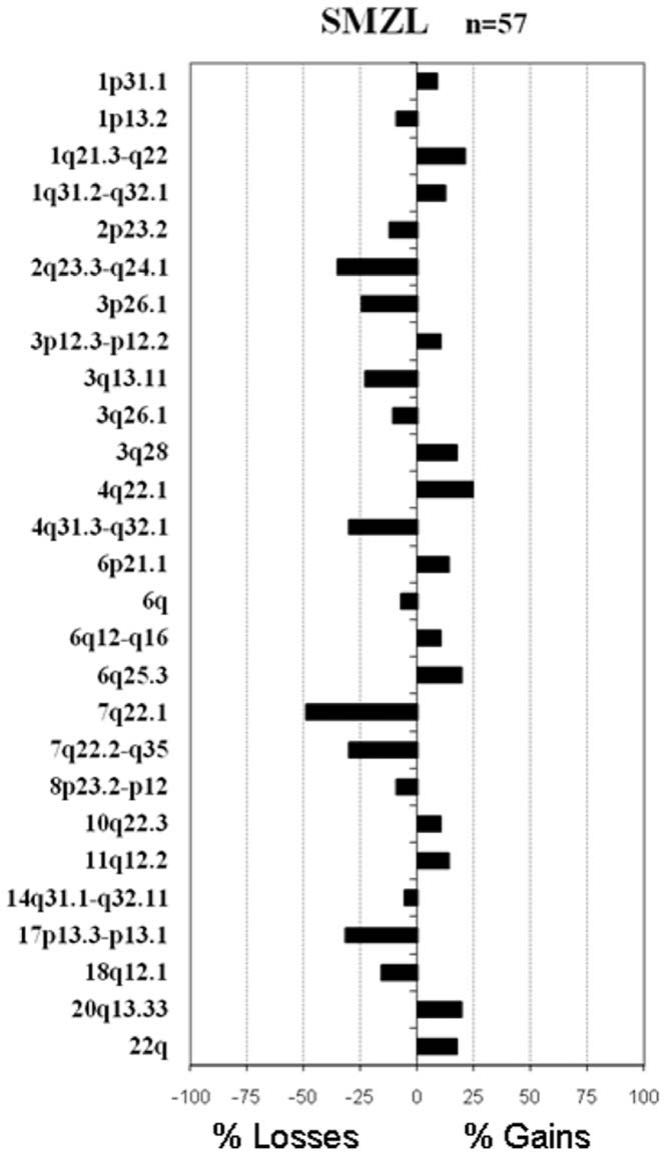
The commonest regions of genomic imbalances as revealed by CGH arrays in splenic marginal zone lymphoma (SMZL). The tree shows the chromosomal regions that exhibited gains (right) or losses (left). For each region, a corresponding cytogenetic location and the respective frequency of change within the cohort are provided.

A total of 73 samples of SMZL were analyzed. Sixty-eight samples were assayed by BAC CGH array and those regions affected by genomic imbalances were annotated for each case. Most of the patients (84%) showed genomic changes. The median number of changes per patient was four (with a range from 0 to 12). The most frequent changes were chromosomal gains involving 4q22.1 (14/57; 25% of patients), 1q21.3–q22 (12/57; 21%), 6q25.3 (11/57; 19%), 20q13.33 (11/57; 19%), 3q28 (10/57; 18%), 22q (10/57; 18%), 6p21.1 (8/57; 14%), and 11q12.2 (8/57; 14%) while the genomic losses were located on 7q22.1 (28/57; 49%), 2q23.3–q24.1 (20/57; 35%), 17p13.3–p13.1 (18/57; 32%), 4q31.3–q32.1 (17/57; 30%), 7q31–q35 (17/57; 30%), 3p26.1 (14/57; 25%), 3q13.11 (13/57; 23%) and 18q12.1 (9/57; 16%) (Fig. 1). The analysis performed by BAC CGH array in SMZL did not identify any homozygous loss in the 7q22.1 region.

### Oligonucleotide CGH array

In addition to the FISH studies, an oligonucleotide CGH arrays analysis was carried out in a group of 19 SMZLs from the global series. Overall, the results provided by the two platforms used confirmed the BAC CGH array results. Thus, alterations such as gains on chromosomes 3, 5q13.2, 6p22.1–p21.1, 8q, 17q and 18, and losses in 4q28.3–q31.23, 10q24.33–q25.3, 15q15.1–q21.1 and 17p13.3–p13.1 were observed with the three CGH array methods.

### FISH validation of losses identified by BAC CGH array

To confirm the genetic imbalances on 7q revealed by BAC CGH array, FISH experiments were carried out in a total of 20 patients. In all cases FISH analysis confirmed the BAC CGH array results. For this purpose, FISH studies in twelve patients, seven of whom had losses in 7q revealed by BAC CGH array and five who had no genetic imbalances in 7q, were performed. FISH confirmed the losses on 7q22.1 previously assessed by BAC CGH array (Table 1). FISH analysis of the 7q33.1 region was performed in eight cases, six of which showed loss of this region with BAC CGH array and FISH studies confirmed the presence of one hybridization signal in these cases. The remaining two cases did not show 7q33.1 abnormalities with either the BAC CGH array or FISH.

**Table 1:** BAC Clones located in the 7q22.1 region.

BAC clone	Position (bp)		FISH Alteration	BAC CGH array
	Start	End		
bA506M12	99507535	99508053	No data	Normal
bA44M6	99873610	100038722	Loss	Loss
dJ1059M17	100976355	101150307	No data	Loss
bA333G13	101175494	101390682	Loss	Loss
bA401L13	102514284	102705988	No data	Normal

### High-resolution analysis of chromosome 7q

**Figure 2:**
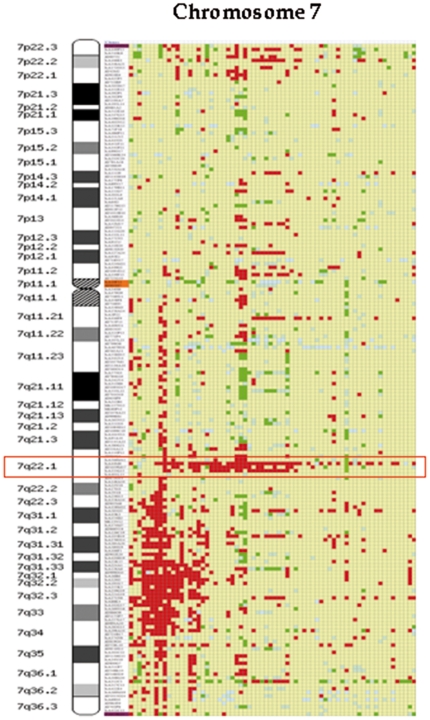
BAC CGH array analysis of chromosome 7 from 68 patients with SMZL. Each patient is shown in columns while the location of the BAC clones is shown in rows. Red colour indicates the presence of a loss, while green indicates a gain of genomic material. Yellow represents a normal amount of DNA, and blue indicates a deficient hybridization. The common deleted region on 7q22.1 is shown in a red box.

Interestingly, BAC CGH arrays detected two regions of losses on chromosome 7q (Fig. 2). One of the regions was located in the cytoband 7q22.1 and was deleted in 49% of patients with SMZL. This region was 1.51 Mb long, located between the BAC clones bA44M6 and bA333G13. The second region was larger, and localized between the 7q31 and 7q35 cytobands. This region was 43.15 Mb in size and situated between the BAC clones bA5N18 (7q31.1) and dJ558L10 (7q35). This second region was less frequent, affecting 30% of SMZL patients, and was located on 7q31–q35. In addition, one patient showed a deletion of only the 7q32 region.

**Figure 3:**
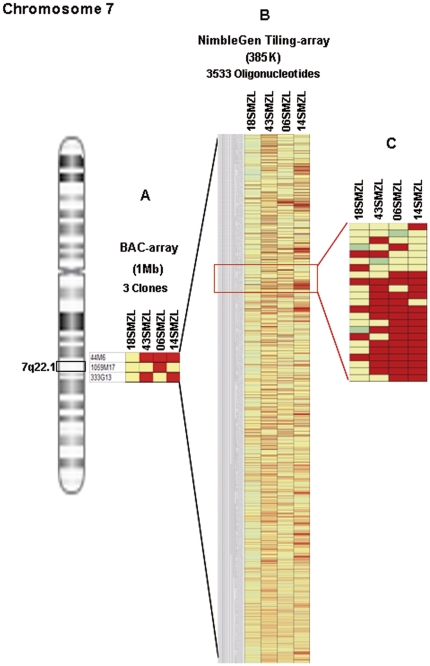
Detailed analysis of the commonly deleted region at 7q22.1 by use of two different CGH arrays. Three cases showed loss in the region (43SMZL, 06SMZL and 14SMZL) between 99925039–101348479 bp (size 1.5 Mb), while the CGH array did not reveal any abnormality of the 7q22.1 region in the control SMZL (18SMZL). (Red, losses; green, gains; yellow, normal; blue, non-informative oligonucleotide hybridization). Figure 3C shows a close-up of a selected region, illustrating the loss on 7q22.1 in three SMZL patients.

Chromosome 7 tiling-array analysis enabled the boundaries of rearrangements found by CGH array to be refined. Chromosome 7 tiling array NimbleGen 385K was used in four SMZL patients. We found three patients with losses on band 7q22.1, where genes such as *MUC3*, *MUC12* and *CUX1* were localized. This focused array was used to obtain a better analysis of the deleted region on 7q. Figure 3 A shows a probe-level view of (A) the BAC CGH array, (B) the CGH array NimbleGen 385K and (C) illustrate the detail of the area of the deletion (7q22.1). The focused array has more probes covering the same genomic area, so it enables a better and more accurate estimate of the exact break-point boundaries compared with the whole-genome array.

### Determination of the commonly lost region on chromosome 7

**Figure 4:**
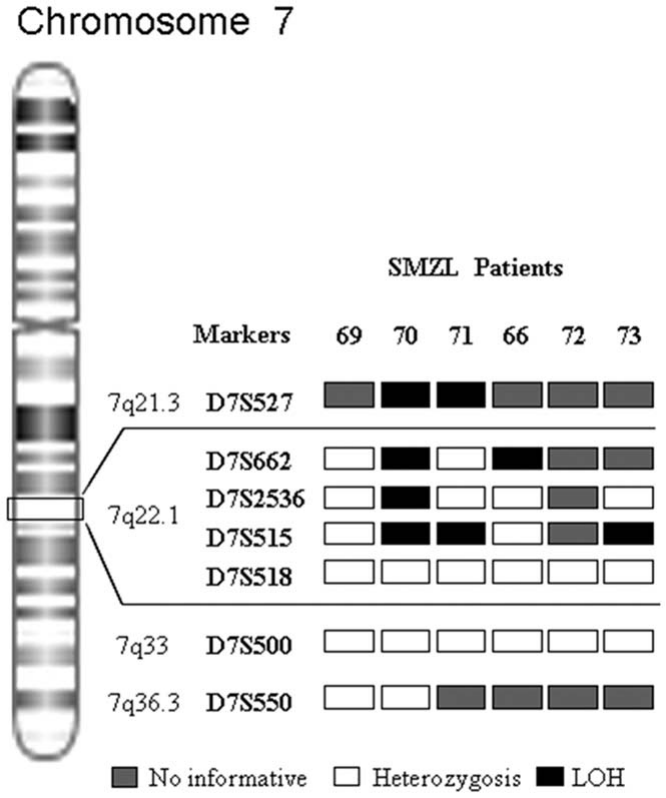
Mapping of LOH in region 7q. (Left) Representation of human chromosome 7, including band assignments. (Right) Names of polymorphic markers used and chromosome band assignments. Four of the six patients analyzed (70, 71, 66 and 73) show LOH for one or more of the markers included in the region 7q22.1 (D7S662, D7S2536, D7S515).

**Figure 5:**
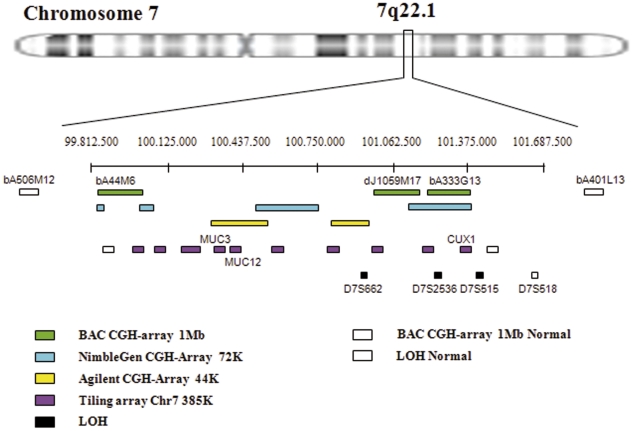
Genetic analysis combined in cytoband 7q22.1 in splenic marginal zone lymphoma. Each method used to analyze the 7q22.1 region commonly deleted in the SMZL is represented by a separate colour. The green boxes correspond to losses detected by BAC mapping to this region; the blue, yellow and purple boxes represent the fragments of the chromosome 7q22.1 region in which losses were detected by commercial NimbleGen arrays. Black boxes show the losses observed with LOH. White boxes define the position of the BAC clones, oligonucleotides and markers of LOH that were not deleted in SMZL.

We analyzed the extent of the region identified on 7q22.1 by the CGH array and tried to define the minimum region of molecular allelic loss. Nine markers were analyzed in six patients with SMZL. The chosen markers have been described in previously published studies of LOH on chromosome 7q [17], [22], [23]. Four of the six patients analyzed (67%) showed LOH on various markers analyzed on chromosome 7. Likewise, the LOH revealed that the markers D7S662, D7S2536 and D7S515, located at 100950585, 101235892 and 101490495, had losses of 50%, 20% and 60%, respectively (Fig. 4). Therefore, based on the analysis of the CGH array and LOH, the region commonly lost in SMZL is located between 99925039 and 101348479 bp (Fig. 5).

## Discussion

The present study assessed DNA copy number alterations in SMZL by using a CGH array and refined the region lost at 7q. In addition to the previous region located at 7q32, the results showed the presence of another small lost region located on 7q22.1. Previous studies using chromosome banding, FISH, LOH, chromosomal-based CGH and oligonucleotide CGH arrays have shown that the partial deletion of the long arm of chromosome 7 is a recurrent abnormality in SMZL [5], [6], [12], [13], [17]–[21], [24], [25].

CGH array results from three different platforms confirmed that partial loss of 7q is among the most frequent imbalances in SMZL. It should be noted that this deletion was precisely delimited in two regions: 7q22.1 with 1.51 Mb size and another well-known region from 7q31 to 7q35 spanning 43.15 Mb. Interestingly, we found the deletion on 7q22.1 by BAC array in 49% of the SMZL samples. This region has usually been out of the lost region on 7q in most of the previous studies [5], [6], [11], [13], [19], [20], [26]–[29]. In our study the BAC CGH array approach, with a resolution of 1 Mb, showed that the deletion on 7q22.1 was delimited by two BAC clones, bA44M6 and bA333G13, and involved *MUC3*, *MUC12* and *CUX1* genes. This recurrent abnormality suggests that this region may contain genes involved in SMZL that are yet to be identified. The genes of the MUC family, which includes *MUC3* and *MUC12*, code for transmembrane mucins, and are involved in epithelial cell protection, adhesion modulation, and signalling; their aberrant expression could be associated with human cancers [30], [31]. The protein encoded by the *CUX1* gene is a member of the homeodomain family of DNA-binding proteins. It may regulate gene expression, morphogenesis and differentiation, and may also play a role in cell-cycle progression. Moreover, it has a function as a transcriptional repressor of the *c-MYC* proto-oncogene. The chromosomal position and function of *CUX1* suggest that it may act as a tumour-suppressor gene [22]. This gene regulates normal B lymphopoiesis, and its alteration is associated with lymphoid abnormalities in mice [32], [33]. Aberrant expression of *CUX1* could be related to tumour progression in a number of cancers [22], [23], [34]–[40], but to date studies of SMZL have not demonstrated any relationship between *CUX1* and this NHL.

In a previous study, 13 markers covering the region 7q21–7q36 were analyzed. The marker D7S487 located on 7q31.3 was frequently lost (45%), while D7S518 (7q22.1) did not show LOH in the cases analyzed [17]. Our study confirmed that the marker D7S518 was heterozygous in all the patients analyzed. However, a more detailed analysis of the 7q22.1 region showed LOH in markers D7S527 (7q21.3; 2 patients), D7S662 (7q22.1; 2 patients), D7S2536 (7q22.1; 1 patient) and D7S515 (7q22.1; 3 patients). It is notable that two of the markers used in the LOH analysis are in the *CUX1* gene located in this region (7q22.1): *CUX1* D7S515 (intron 3) and *CUX1* D7S518 (intron 20) [22]. Therefore, the LOH complements the findings obtained from the CGH array, which revealed a frequent loss of genomic material in the 7q22.1 region. When we analyzed this region in greater depth, through tiling arrays and LOH studies, it was possible to refine the mapping of the region to Chr7: 99925039–101348479 bp.

CGH arrays showed a second region, located on 7q31–q35, that has been found to be deleted in SMZL in previous studies [5], [6], [12], [13], [17]–[21], [25], [24]. This region was 43.15 Mb in size, ranging between the BAC clones bA5N18 and dJ558L10. In the present study the region was lost in 30% of SMZL patients. This anomaly is considered a relatively specific genetic marker of SMZL and has been associated with an aggressive clinical course [17]. The region includes genes such as *THAP5*, *IMMP2L*, *FOXP2*, *TES*, *MET*, *ST7*, *PTPRZ1*, *GRM8*, *UBE2H*, *MLKN1*, *BPGM*, *CALD1*, *PTN*, *SVOPL*, *AGK*, *KEL*, *TPK1* and *CNTNAP2*. The *MET* gene is a proto-oncogene and an important regulator of cell proliferation and differentiation, organ regeneration, embryogenesis and oncogenesis. It may be one of the longest-sought oncogenes controlling progression of primary cancers to metastasis. By contrast, the *ST7* gene is a tumour suppressor gene that is underexpressed in mantle cell lymphoma [41].

In summary, the resolving power of the CGH array enabled us to confirm that the most frequent alterations in SMZL were losses on chromosome 7, and to identify a new region in the band 7q22.1 (between 99925039–101348479 bp).

## Materials and Methods

### Patients

Seventy-three patients with the diagnosis of SMZL were studied. All cases were reviewed by expert haemopathologists (TF, MM, MAP) and the diagnosis was established according to the WHO classification [3], [42], [43] Forty of the patients (52%) were women. The ages ranged from 44 to 86 years (median 69 years). The study was approved by the local ethical committees “Comité Ético de Investigación Clínica, Hospital Universitario de Salamanca”. Informed consent was obtained from each patient before they entered the study.

### Methods

Of the 73 patients with SMZL included in the study, 68 were analyzed by BAC CGH arrays. To confirm the results from these, commercial oligonucleotide microarrays were used in 19 of these patients (a NimbleGen CGH array 72 k was used for 11 patients, while the remaining eight were analyzed with an Agilent CGH array 44 k). Four of the 68 cases were studied by tiling array. Six cases were studied by LOH, five of which were not studied by BAC CGH arrays. The array data has been deposited in the gene expression omnibus database, the accession number is GSE31203.

### DNA isolation

All genomic DNA was extracted from fresh-frozen samples using the standard phenol-chloroform method. Tumour DNA was isolated from the spleen (49 cases), peripheral blood (27 cases) or bone marrow (3 cases). Of the 68 cases analyzed using the BAC arrays, most (44 patients) were studied in spleen while the remaining 24 were analyzed in PBL. In all samples the tumour cell percentage was greater than 50%. Normal DNA was extracted from the human placenta of healthy donors. All DNA was quantified using a NanoDrop spectrophotometer (ND-1000, NanoDrop Technologies, Wilmington, DE). DNA quality was assessed by the 260∶280 ratio and its integrity by agarose gel ethidium bromide visualization [44].

### CGH array studies

#### 1. BAC CGH array

A genome-wide analysis of DNA copy number changes of patients was performed using a BAC CGH array. Slides containing 3528 BACs spanning the genome were produced at the Cancer Research Centre (Salamanca, Spain) as previously described [45]. Briefly, to test for labelling reactions, 2 µg of unamplified genomic DNA, test (tumour DNA) and reference material (placental DNA) were digested separately with *DpnII* restriction enzyme (New England Biolabs, Beverly, MA) and separately labelled using random primers (BioPrime Labeling System, Invitrogen, Carlsbad, Calif) and Cy5-dCTP and Cy3-dCTP (CyDye™ 3-dCTP and CyDye™ 5-dCTP, Amersham Biosciences, Piscataway, NJ) fluorescent dye for paired hybridization samples, respectively. The incorporation of labelled nucleotide was quantified using a NanoDrop spectrophotometer (ND-1000, NanoDrop Technologies). Labelled test and reference DNA samples were mixed equitably, co-precipitated in the presence of Cot-1 human DNA (Roche, Indianapolis, IN) with ethanol, washed, and resuspended in hybridization solution (50% formamide, 10% Dextran sulphate, 2× standard saline citrate, 10 mM Tris pH 7.6, 2.7% sodium dodecyl sulphate and 10 µg/µl of yeast tRNA). DNA mixtures were cohybridized to the arrays in a GENETAC (Genomic Solutions, Ann Arbor, Michigan, USA) for 48 hours at 42°C in accordance with the manufacturer's recommended protocol.

Images and signal intensities were acquired using a GenePix 4000B (Axon Instruments, Burlingame, CA) dual laser scanner in combination with GenePix Pro4.0 (Axon Instruments, Burlingame, CA) imaging software. Fluorescence ratios were normalized using the median of the fluorescence ratios of every spot, computed as log_2_ values. The log_2_ ratio of each clone was normalized to the median log_2_ ratio of the 20 control hybridizations, after which the median of the three spots was calculated. Data from two-colour hybridizations for both sets of DNA were normalized using the DNMAD module of the GEPAS software [46]–[48]. Regions of copy number gain and loss for the BAC CGH array data were identified by creating sample-specific thresholds [49]. A cut-off value of 0.4 was used, based on the ratios of clones in ten hybridizations of normal male versus normal female DNA [45]. The clones with log_2_ ratios above or below a control sample's threshold value were considered as gains or losses, respectively. At least two contiguous BAC clones with a log_2_ ratio of −0.4 or less were defined as a region of loss and a log_2_ ratio of +0.4 or more was defined as a region of gain. Furthermore, spots with weak Cy3 or Cy5 intensity (R^2^<0.2) and clones with a standard deviation of more than 0.3 from measurement of the three spots were excluded from the analysis. Approximately 10% of clones were excluded in this way. All data sets were carefully reviewed for frequently affected chromosomal sites of physiological copy number polymorphisms (CNPs). Every clone on the array was compared with the ‘Database of Genomic Variants’ (available at: www.project.tcag.ca/cariation; accessed November 2009) and that of chromosomal imbalances and phenotype in humans using Ensembl Resources (DECIPHER: available at: http://www.sanger.ac.uk/PostGenomics/decipher/; accessed November 2009) [49]–[51].

#### 2. Oligonucleotide CGH array platforms

To confirm the results from the BAC CGH array analysis, two types of oligonucleotide CGH array platforms were used. Eight SMZLs were analyzed with Agilent's Human Genome CGH Microarray 44 k (Agilent Technologies). The microarray contains 44255 *in situ* synthesized 60-mer probes spaced at 43000 bp intervals throughout the human genome and includes 3877 controls. The probes are, according to the manufacturer's description, enriched in cancer-relevant genes, representing both coding and non-coding sequences on the chromosomes. Experiments using Agilent arrays were performed using human placenta genomic DNA as reference and following Agilent's recommended standard protocol. The arrays were scanned using the Agilent scanner, and data were extracted, filtered and normalized using the Feature Extraction program (Agilent Technologies). The Agilent CGH Analytics Software 3.4 trial (Agilent Technologies) was used to export the CGH array data for use with Nexus Copy Number Professional trial software (version 4, BioDiscovery Inc) [52].

Eleven patients with SMZL were analyzed with NimbleGen human CGH 4×72K Whole Genome v2.0 array (Roche NimbleGen, Inc). Placenta DNA was used as the reference. The CGH array protocol from NimbleGen Systems was followed. Briefly, 500 ng of tumour and reference DNA were labelled with Cy3 and Cy5, respectively. Labelled material was co-hybridized to microarrays consisting of 71341 oligonucleotide probes spaced at approximately 40000 bp intervals throughout the human genome, washed and scanned at 10 µm resolution using the GenePix 4000B dual scanner (Axon Instruments, Burlingame, CA). Raw data were extracted using NimbleScan software v2.5 (Roche NimbleGen, Inc), which enables automated grid alignment, extraction and generation of data files [53].

#### 3. Detailed analysis of chromosome 7

*Fluorescence in situ hybridization (FISH) analysis.* To confirm the losses identified by CGH array, FISH analysis was performed using the BAC clones bA44M6 mapped to 7q22.1 (99873610–100038722 bp), bA333G13 mapped to 7q22.1 (101175494–101390682 bp), bA36B6 mapped to 7q31.31 (130078078–130270796 bp) and bA371N6 mapped to 7q33 (134684519–134842787 bp) as previously described (NCBI36/hg18) [54]. These clones were selected from the same BAC CGH array clones library used for the BAC CGH array studies (Wellcome Trust Sanger Institute, Cambridge, UK). DNA from the BAC clones was isolated and directly labelled with either Spectrum Green-dUTP or Spectrum Orange-dUTP (Vysis/Abbott Molecular, Inc.) by nick translation and hybridized as previously described [54]. All BAC clones were first hybridized to normal human metaphase chromosomes in order to verify their location. A minimum of 200 interphase nuclei were scored using an E1000 microscope (Nikon, Tokyo, Japan) equipped with the Quips system (Vysis, Downers Grove, IL).

*Tiling CGH array.* In order to localize the lost region at 7q better, four patients with SMZL were analyzed using tiling-path CGH arrays. These arrays were designed at higher resolution with fine-tiling analysis of chromosome 7 (chromosome7:117711–158805222 bp). This array was constructed by maskless array synthesis technology (Roche NimbleGen, Inc.), with up to 385110 oligonucleotides, with a median probe spacing of 365 bp, and synthesized by photolithography on an array by previously described methods [55], [56]. Arrays were scanned at 5 µm resolution using the NimbleGen MS 200 Microarray scanner (Roche NimbleGen, Inc) data were extracted from the scanned images using NimbleScan software v2.5 (Roche NimbleGen, Inc) [53].

*LOH analysis.* DNA from patients was extracted from spleen and peripheral blood or bone marrow tissues as previously described. Seven microsatellite markers on chromosome 7 were used (Table 2). Four of these markers, D7S662, D7S2536, D7S515 and D7S518, were used to define more precisely the level of the deleted region 7q22.1 detected in a high percentage of SMZLs. All microsatellite markers were selected on the basis of their location (Genethon map release, GDB and RHdb) and on the frequent LOH detected in losses on 7q. PCR reactions were performed in a final volume of 25 µl containing approximately 30–50 ng/µl of template DNA, 5× PCR buffer, 2.5 mM dNTPs, 25 mM Mg_2_Cl, 10 µM of each primer, and 1 U of Taq DNA polymerase (Promega, Madison, WI, USA). PCR was performed in a thermal cycler. Amplification consisted of an initial denaturing step of 5 min at 94°C, 35 cycles of 30 s at 94°C, 30 s at 55–65°C and 30 s at 72°C, followed by a final step of 5 min at 72°C. The PCR products were separated by 3% of agarose gel (MS4 Metagel, Conda).

**Table 2:** Microsatellites markers selected for the LOH analysis.

Markers	Region Chromosome	Size (bp)	Localization (bp)		Sequence
D7S527	7q21.3	273–297	95453048	Forward	CATTGCAAACTCAGGAGATA
				Reverse	TAACAGAGGCATGAAAACCA
D7S662	7q22.1	204–234	100950585	Forward	GTTGACAGACAAGCACAGAC
				Reverse	AGCTGTTTCCCATTTCCA
D7S2536	7q22.1	118–141	101235892	Forward	ACACTCCGCCACCTTG
				Reverse	CAACAACTGTTCCTAAAGCCT
D7S515	7q22.1	128–190	101490495	Forward	GGGAGTTACTACCCTCACTTAATG
				Reverse	GGACTGGGCAGCAAAG
D7S518	7q22.1	179–201	101648938	Forward	CAGTAGGCAGGGGTGG
				Reverse	GGGTGTGTCTGTGTGACAAC
D7S500	7q33	188–210	134759259	Forward	CCAGAATTGAAAACTCAGCA
				Reverse	ATTGATTGAGGAACTGAACTTACCT
D7S550	7q36.3	177–200	155210270	Forward	TCTCATCTGTGAATGCACTATC
				Reverse	GCAGTTGGGTTATTTCAAGTC

Allelic loss was scored only for informative patients whose normal DNA samples were polymorphic at a given locus. LOH was identified by visual analysis as a loss in intensity or complete loss of one allele in the tumour DNA when compared with the normal DNA from the same patient. All cases of LOH were agreed by three reviewers.
